# New Application of Osteogenic Differentiation from HiPS Stem Cells for Evaluating the Osteogenic Potential of Nanomaterials in Dentistry

**DOI:** 10.3390/ijerph17061947

**Published:** 2020-03-16

**Authors:** Giulia Tetè, Paolo Capparè, Enrico Gherlone

**Affiliations:** 1 Specialization School in Oral Surgery, Vita-Salute San Raffaele University, 20132 Milan, Italy; 2 Dental School, Vita-Salute University and IRCCS San Raffaele, 20132 Milan, Italy; gherlone.enrico@hsr.it

**Keywords:** stem cells, nanomaterials, biomaterials, bone substitutes, growth factor, bone regeneration

## Abstract

Objective: HiPS stem cells are commonly used for the study of medical disorders. The laboratory in which this study was conducted uses these cells for examining the treatment and cure of neurodegenerative diseases. Bone regeneration poses the greatest challenge for an oral surgeon both in terms of increased implant osseointegration and reducing bone healing times. The aim of this study was to validate the protocol in the literature to produce and then test in vitro osteoblasts with different nanomaterials to simulate bone regeneration. Method: hiPS clones (#2, #4, and #8) were differentiated into an osteoblast cell culture tested for alizarin red staining and for alkaline phosphatase testing at 14, 21 and 28 days, after the cells were plated. Results: The cells showed diffuse positivity under alizarin red staining and the alkaline phosphatase (ALP)-test, showing small formations of calcium clusters. Conclusion: Despite the limitations of our study, it is a starting point for further protocols, laying a solid foundation for research in the field of bone regeneration through the use of stem cells.

## 1. Introduction

Tissue engineering will play a key role in applications in oral bone defects caused by edentulism, trauma, cancer, and other diseases [1] In the past, bone defects have been treated with bone substitutes; i.e., heterologous, homologous, alloplastic, and autologous bone, the last of which has been the gold standard for substitutes as it is characterized by osteogenesis, osteoinduction, and osteoconduction. However, it is an invasive method for the patient and it is surgeon-dependent [2,3].

The physical and chemical properties of nanomaterials lead to microcharacteristics increasing the contact surface with the biological material being regenerated [4]. The low porosity of nanomaterials allows the conveyance of growth factors, proteins, drugs, and oligonucleotides to the tissue of interest [5]. Therefore, in bone engineering, nanomaterials are being considered for their excellent physical, chemical, and biocompatibility properties [6]. Nano-hydroxyapatite is a material that, combined with polymers such as collagen, polylactic acid (PLA), poly lactideglycolic acid (PLGA), polyamide, coralline, chitosan, and polycaprolactone (PCL), can be used in tissue engineering, especially in bone regeneration [7].

Stem cells are already used in tissue engineering and bone regeneration. In combination with biomaterials that guide them in differentiation, they are especially used as bone grafts [8]. Yamanaka discovered the totipotent proliferative capacity of cells reprogrammed with viral factors OCT4, SOX2, KLF4, and c-Myc; such cells became the induced pluripotent human stem cells (iPSCs) [9]. This type of cell has already been used in the study of diseases and in pharmacology, such as in the field of neurodegenerative diseases [10].

Embryonic stem cells (ESCs) and mesenchymal stem cells (MSCs) are safer than iPSCs due to the lower risk of carcinogenesis and hyperproliferation; however, they have less proliferative capacity and more epigenetic memory [11]. Several scaffolds have been studied to complete the osteogenic differentiation of iPSCs with bone grafts, but with limited results; nanomaterials and biomaterials should be tested with iPSCs first regarding their differentiation in an osteogenic sense [12].

The purpose of this study was to formulate a protocol for osteogenic differentiation from stem cells to evaluate the osteogenic potential of nanomaterials in dentistry using alizarin red staining and alkaline phosphatase (ALP). Such findings will provide innovative knowledge in the field of bone regeneration with further clinical applications for biomaterials and stem cells (i.e., alveolus regeneration).

## 2. Materials and Methods

### 2.1. iPSC Reprogramming, Characterization, Validation of Pluripotency, and Multilineage Differentiation Potential

Three human iPSC clones (#2, #4, and #8) were generated by reprogramming healthy subject fibroblasts using Sendai virus technology (CytoTune-iPS Sendai Reprogramming Kit, ThermoFisher, Waltham, MA, USA) [13].

HiPS clones were individually picked and expanded on a feeder layer in mTeSR1 medium (STEMCELL Technologies, Vancouver, BC, Canada). Cells were maintained in mTeSR1 on hESC-qualified Matrigel (BD Biosciences, Franklin Lakes, NJ, USA), dissociated with 0.5 mM EDTA (Ethylenediaminetetraacetic acid) (Ambion, Waltham, MA, USA) for passages, and were routinely tested for mycoplasma.

iPSCs cultured on Matrigel ES (Sigma-Aldrich, Sant Louis, MO, USA) were treated with 0.2 mg/mL colchicine for 16 h and sent to Integrated System Engineering (ISENET, Milan, Italy) for karyotype analysis using a Q-banding on 400 bands and by array cytogenetic hybridization (aCGH) of 600,00 probes with a median probe spacing of 41 kb (Agilent Technologies, Santa Clara, CA, USA). Cells were tested for pluripotency markers using immunofluorescence (OCT4, NANOG, SSEA3, SSEA4, and SOX2) and flow cytometry (SSEA4-FITC, TRA1-60-APC, and TRA1-81-APC; Millipore, Burlington, MA, USA).

To assess the ability of iPSCs to differentiate in the three germinative lineages, iPSC colonies were incubated with Dispase solution (Gibco, Waltham, MA, USA) for 10–15 min at 37 °C to promote colony lifting. Cell aggregates (embryoid bodies, EB)) were maintained in differentiation medium consisting of DMEMF-12, 20% (medium for stem cells) knock-out serum replacement, 20 mM b-mercaptoethanol, 1% sodium pyruvate, 2 mM l-glutamine, 2 mM nonessential amino acids (NEAA), 100 U/mL penicillin, and 0.1 mg/mL streptomycin (Gibco, Thermofisher, Waltham, MA, USA) for 5 days in the presence of ROCK inhibitor (Rho-associated protein kinase inhibitor) (STEMCELL Technologies, Vancouver, BC, Canada).

### 2.2. Differentiation iPSCs in Osteoblast Cells

Human iPSCs were differentiated into osteoblast cells following a protocol established for pluripotent stem cells [14], with slight modifications. Differentiation was initiated in adhesion when the iPSC cultures reached 70–80% confluence.

iPSCs were aspirated and washed with PBS, and then treated with 0.5 mg/mL dispase-DMEM/F12 for about 10–20 min until the colonies dissociated into clumps and departed from the bottom of the plate. We washed them gently with PBS 3 times and resuspended them in EB medium: DMEM/F12, 20% KnockOut™ Serum Replacement (Thermofisher, Waltham, MA, USA), 1:100 P/S (penicillin-streptomycin)(Gibco, Thermofisher, Waltham, MA, USA) 1:100 L/G (CD27 Monoclonal antibody), 1:100 non-essential amino acid (NEAA)(Gibco, Thermofisher, Waltham, MA, USA) Cell Culture Supplement, 1:100 sodium pyruvate, and 1:2500 2-mercaptoethanol. The colonies were transferred to non-adherent 3.5 cm Petri dishes.

After one day, we controlled EB formation and changed media to avoid excess cell death. On day 3, we changed the EB medium; on day 4, we coated cells with 0.1% gelatin MW6 (multiwell six), then we aspirated the gelatin and plate EBs in fresh media, distributing in more wells depending on the amount. When 70–80% confluence was reached, we used Accumax (Sigma-Aldrich, St. Louis, MO, USA) and then a scraper and plate on coverslips with 7 × 10^4^ cells/well with mw24 coated with 0.1% porcine gelatin in EB Medium + Rock.

The day after this, we switched to an osteogenic differentiation medium (DMEM/F12 with 20% (*v*/*v*) FBS, supplemented with 50 μM ascorbic acid, 10 mM β-glycerophosphate, and 10 nM dexamethasone) [15]. After another 2 days, we added retinoic acid to some wells of ctr8 (Sigma-Aldrich, St. Louis, MO, USA). We changed the medium every 2 days. Analyses of differentiation were performed after 14, 21, and 28 days (if possible for the number of cells) using the alizarin red/Von Kossa AP-KIT. We examined the cells using a phase microscope.

### 2.3. Alizarin Red Staining

After 14, 21, and 28 days in osteogenic medium, we performed the alizarin red staining test for all controls; we used 4% paraformaldehyde (PFA, Sigma-Aldrich, St. Louis, MO, USA) solution in PBS (Lonza, Basel, Switzerland) for 10 minutes in a well, and then we washed twice gently with PBS. Then, we maintained 400 μL of alizarin red at room temperature (RT) for 30 min. We washed with 2 mL H_2_0 and then mounted cells with Dako (mounting medium) on a glass side, taking images using an upright microscope at 4× and 20× magnification.

### 2.4. Alkaline Phosphatase

We collected the medium and placed it in a 1.5 mL Eppendorf tube. After we removed the cells and washed them with PBS, we then collected the medium and cells in another Eppendorf at 14, 21, 28, and 35 days after the plate. The samples were stained for ALP activity using an ALP kit (SensoLyte® pNPP alkaline phosphatase assay kit, colorimetric).

All the outcomes were evaluated on the clones reported in Table 1.

### 2.5. Ethical Approval

This study was approved by the I.R.C.C.S. San Raffaele Srl Hospital under registration number BANCA-INSPE version 03 del 9/2/2017.

## 3. Results

**Figure 1:**
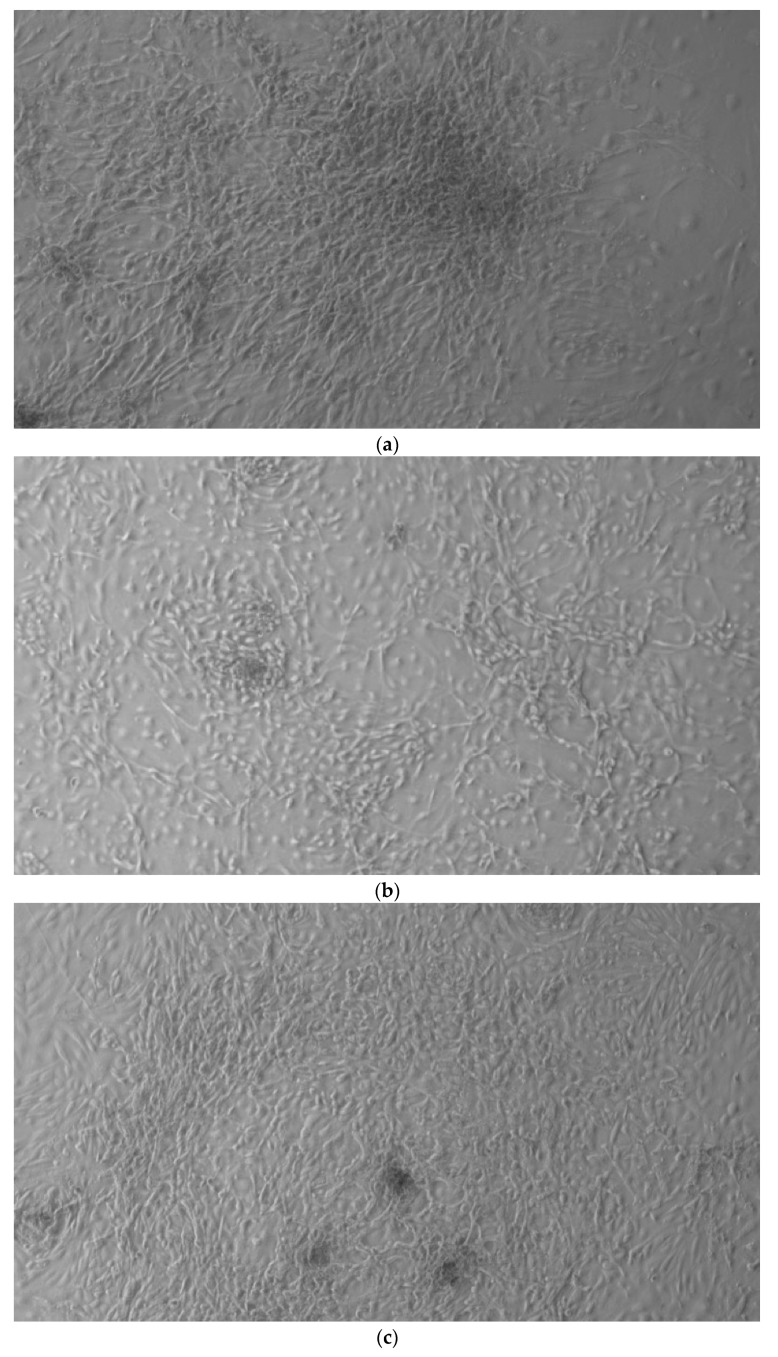

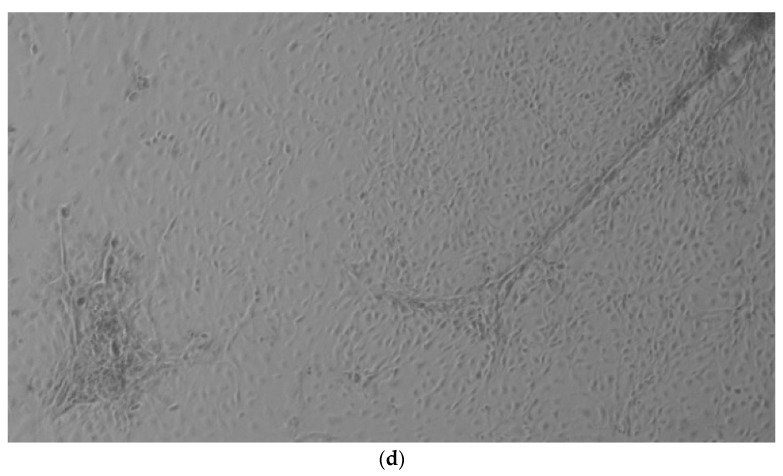
HiPS stem cells are coated as EB ((embryoid bodies) mandatory step before differentiating into osteoblasts), when they reach confluence; i.e., a situation in which they no longer have space to grow They then move on to the phase of differentiation in the osteoblastic sense, thus changing the type of medium. (**a**) CTR2#6 P19 (one of four clones) reached confluence (70–80%) on day 12 (magnification 10×, plates 70,000 cells); (**b**) CTR4#5 P20 reached confluence (70–80%) on day 21 (magnification 10×, plates 70,000 cells); (**c**), CTR8#14 P12 + RA reached the confluence (70–80%) on day 21 (magnification 10×, plates 70,000 cells); (**d**) CTR8#14 P12 - RA reached confluence (70–80%) on day 6 (magnification 10×, plates 70,000 cells).

**Figure 2:**
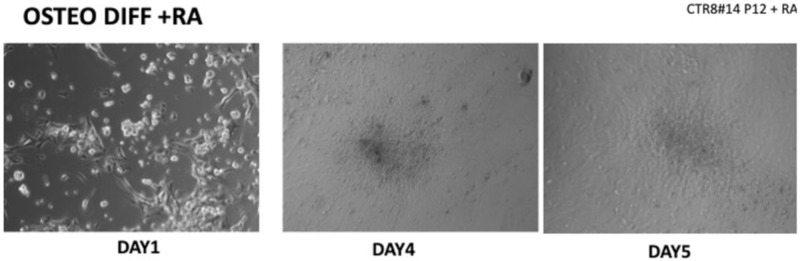
Once the EBs were ready they were plated in an osteogenic differentiating medium that allowed them to mature into osteoblasts; we can see from the images how, from the first days, the cells showed the typical morphologies of osteoblasts. The 8+RA control (cell clone) in the osteogenic differentiation medium initially showed rapid growth, as seen from the optical microscope images. (magnification 20×–10×, plates 70,000 cells). We see clone growth at 1, 4 and 5 days.

**Figure 3:**
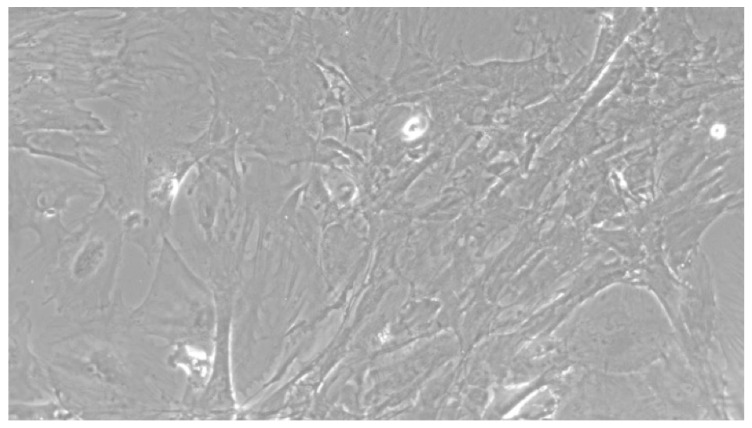
CTR8#14 P12 + retinoic acid showed a slower growth than the start of day 12 of differentiation. In the optical microscope image, in fact, we can see how the cells are organized in the typical osteoblast structure: concentric and tapered. (magnification 10×, plates 70,000 cells).

**Figure 4:**
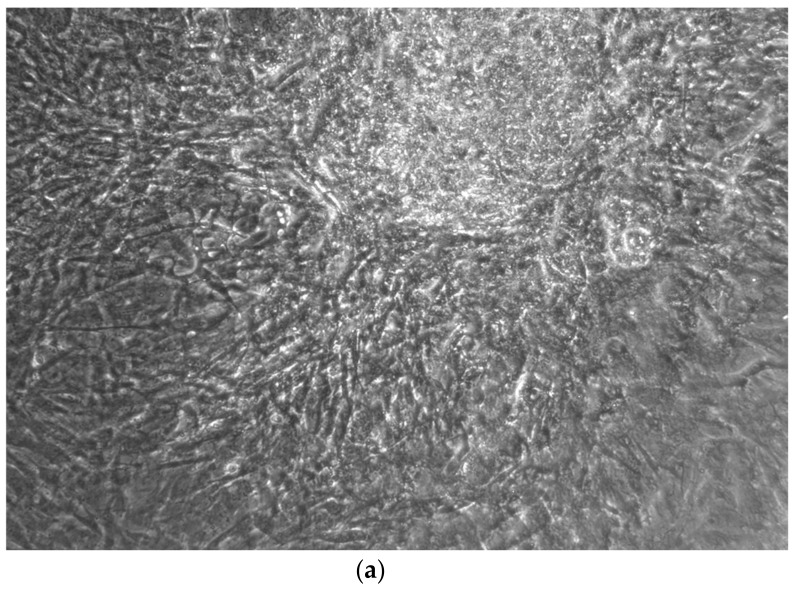

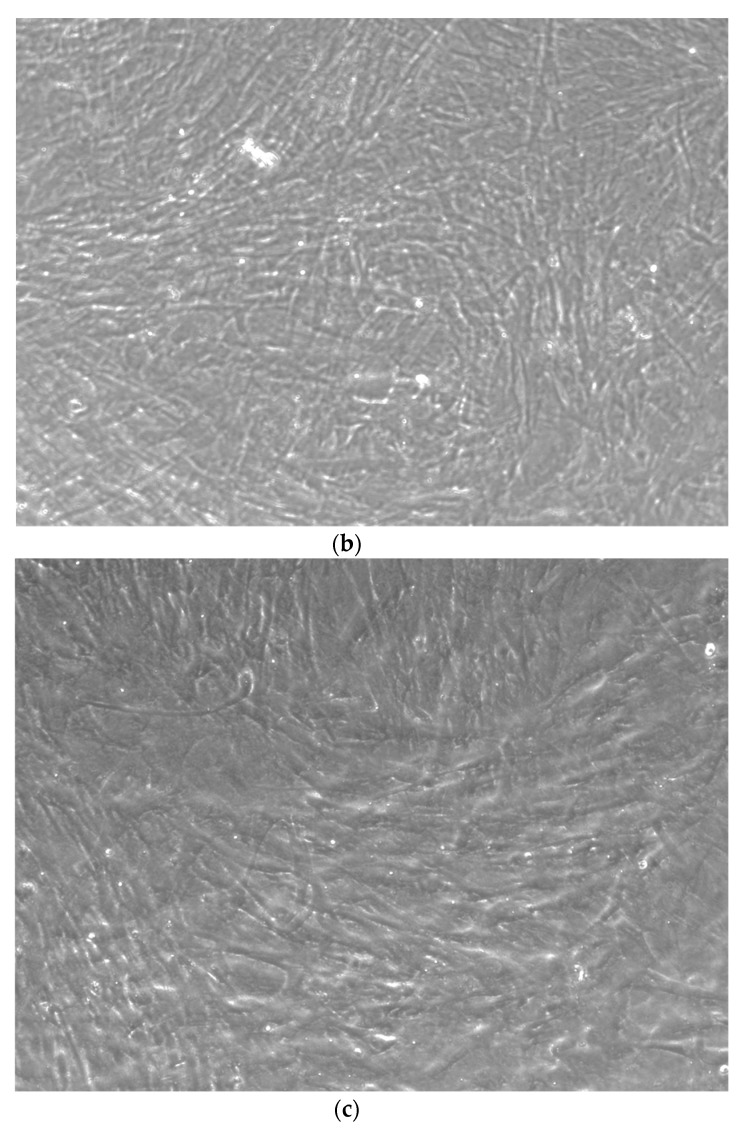
CTR4#5 P20 showed a different structure since the first days of differentiation compared to other clones; these optical microscopic phase images are, respectively, at 7 (**a**), 14 (**b**), 26 (**c**) days and do not show the typical concentric structure of the lamellar bone, but rather a highly disorganized structure.

**Figure 5:**
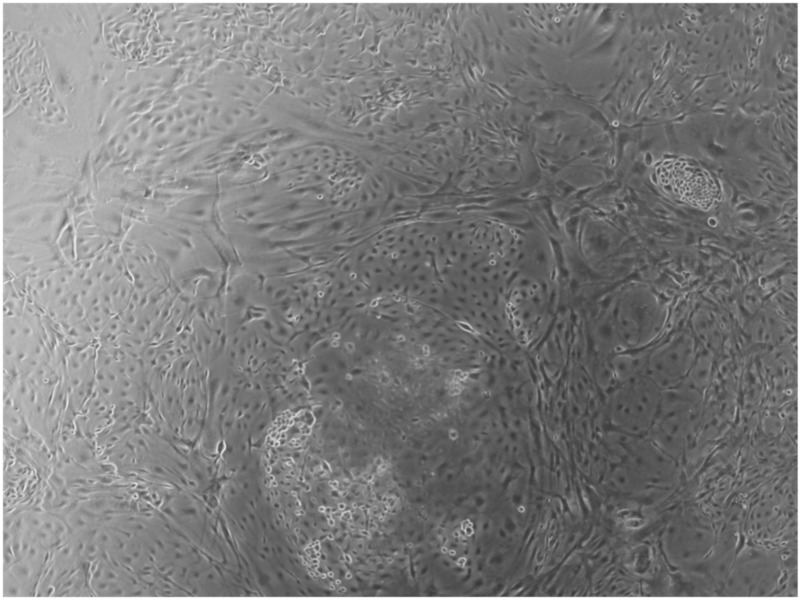
CTR8#14 P12 – RA showed the typical image of bone formation, where the collagen fibers, which make part of the extracellular matrix, begin to be arranged in a concentric way, thus giving a rudimentary appearance to the osteon, but one which is typical of the early stages of osteogenesis.

**Figure 6:**
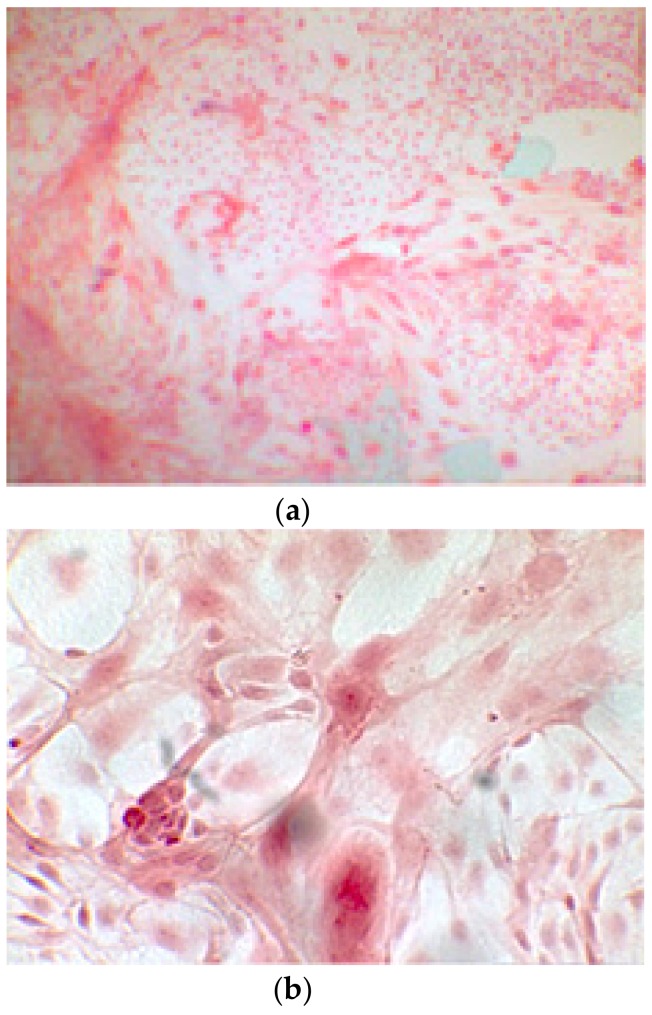

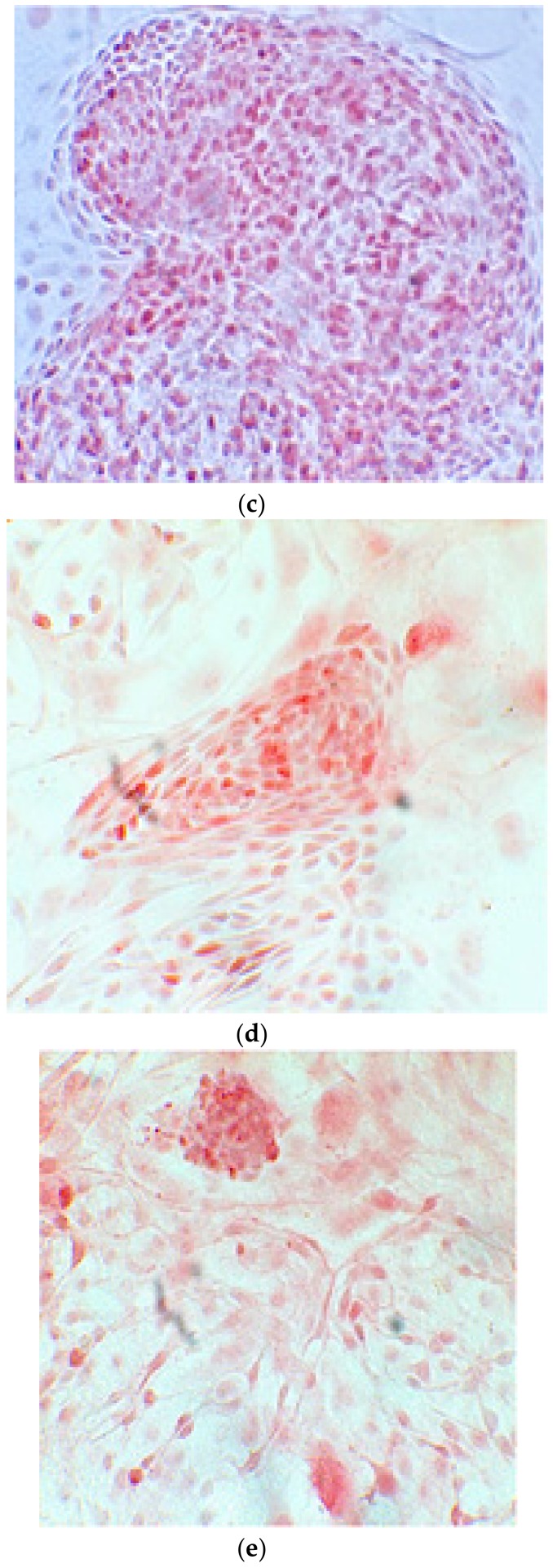

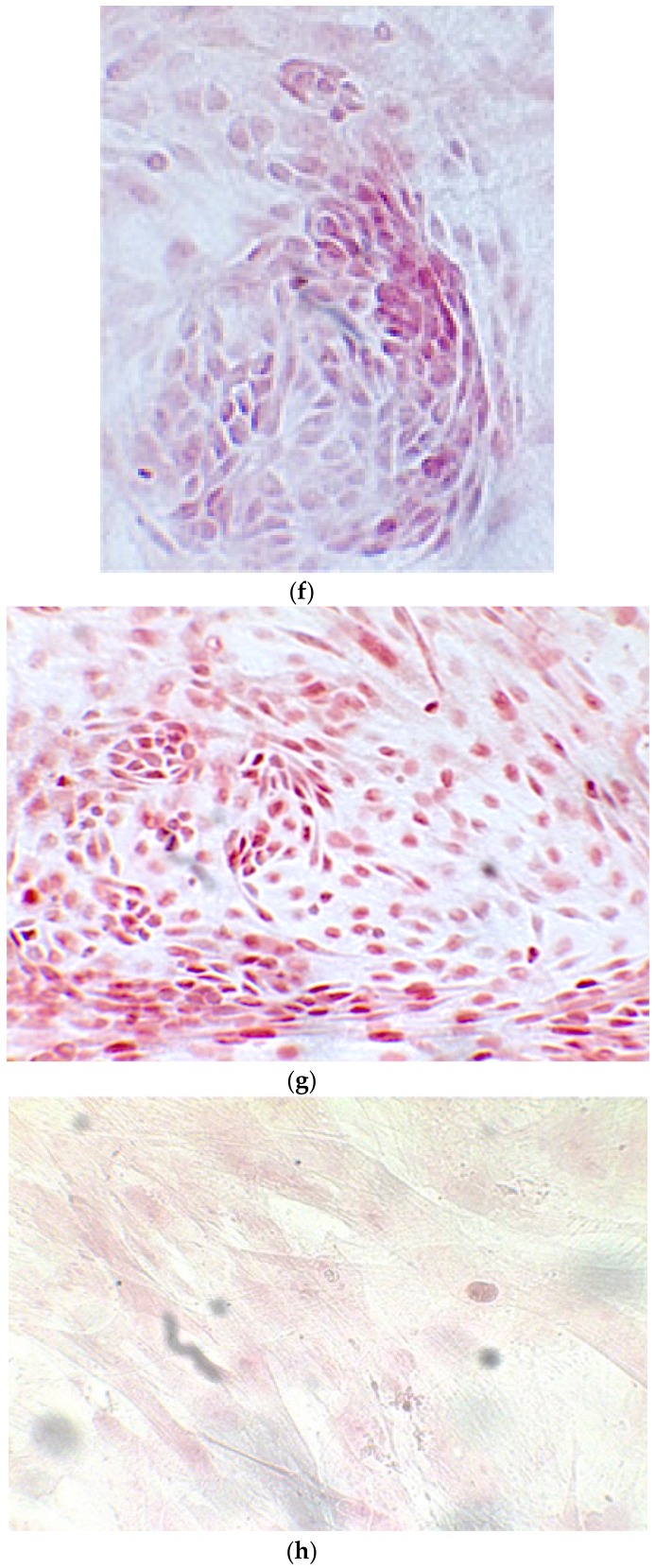
Since the first images processed under the microscope after staining with alizarin, we see that those after 14 days are positive to the test of alizarin, so the partially differentiated cells include calcium. We have one image for control CTR2#6 P19 (**a**), five images for CTR8#14 P12 – RA (**b–f**) control, being the best clone, and one image for control CTR8#14 P12 + retinoic acid (**g**).

When the EBs were coated, confluence was expected at 70–80% (Figure 1a–d); in CTR8#P12, since it was the most copious clone, retinoic acid was added as described previously (Figure 2) [16]. From the phase microscope images, we observed that, in the first days of differentiation, the speed of expansion and differentiation was faster (Figure 3), which then appeared to slow (Figure 4). CTR4#5 P20, as shown in the microscope images (Figure 5), followed a different trend: its structure was more streamlined than the other clones, and even the phyllopod-connecting structures typical of the morphology of osteoblasts appeared different. CTR8#14 P12 without retinoic acid immediately showed a concentric differentiation, then in the typical form of osteoblasts, and then the clone developed real areas of osteogenesis with the formation of osteons, confirmed by alizarin testing and alkaline phosphatase (Figure 6a–c).

### 3.1. Alizarin Red Staining

**Figure 7:**
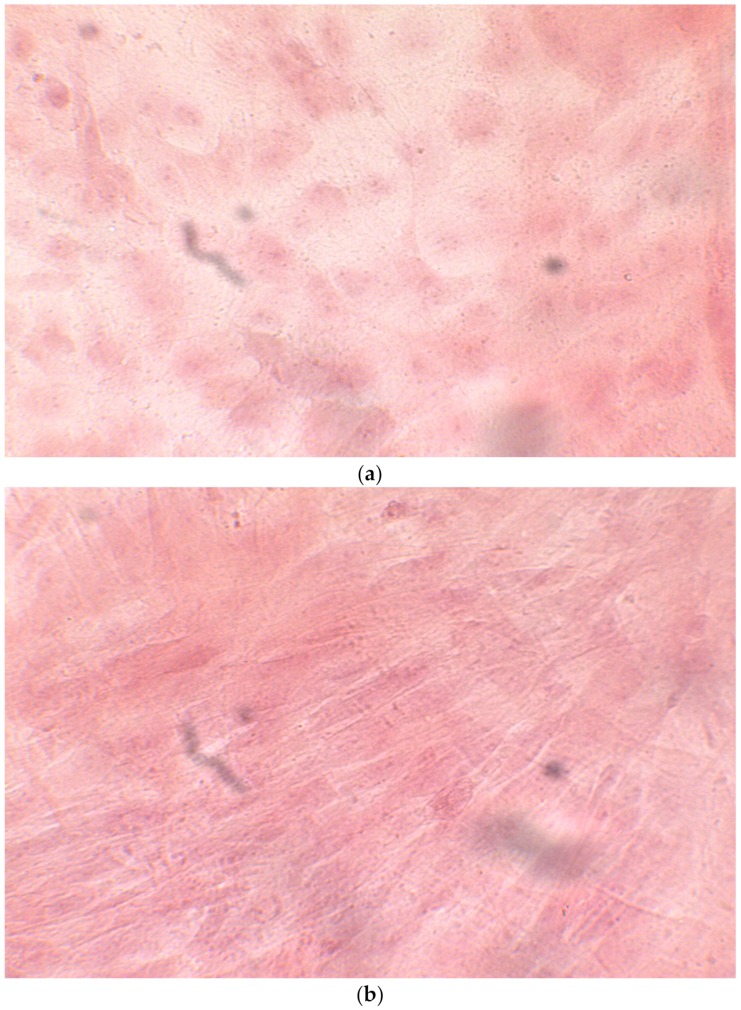
CTR4#5 P20: despite being positive to the alkaline test, as seen in the phase optical microscope, the alizarin red staining test shows a different structure from the other clones that appear more spherical (**a**,**b**).

**Figure 8:**
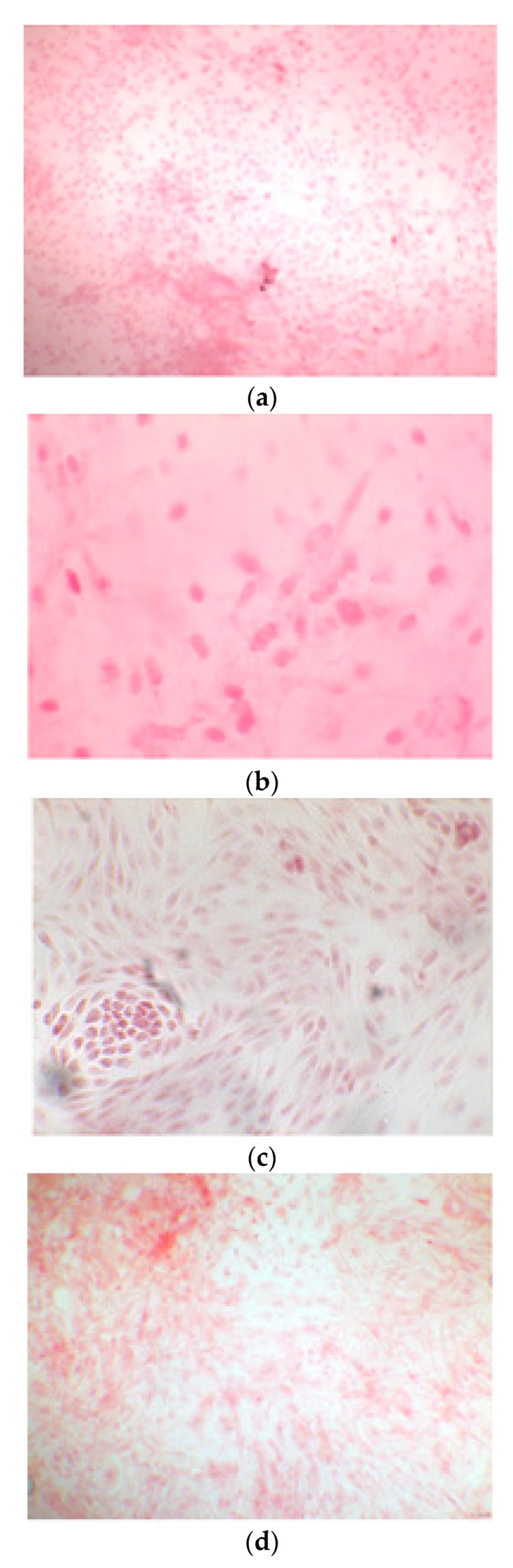

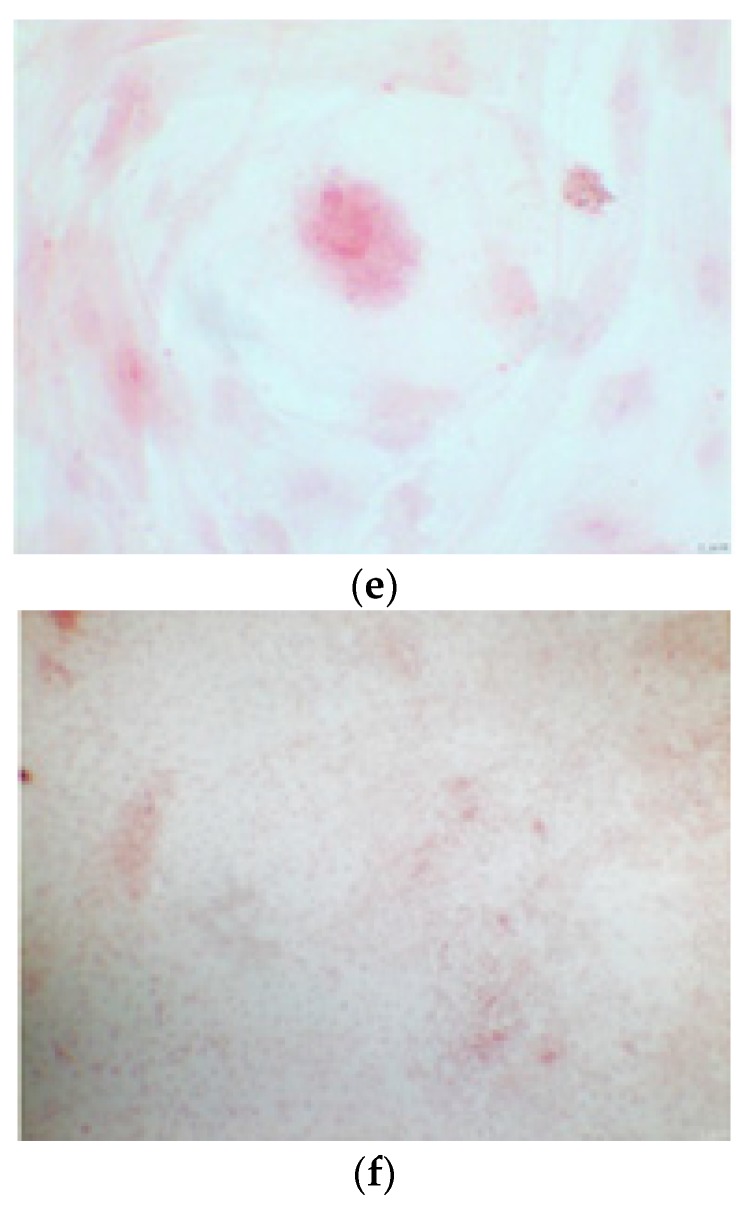
The alizarin test at 21 days confirmed the positivity of samples of calcium deposits, highlighted by red coloration. Compared to 14 days, a more organized structure is evident; we can see this from a smaller red coloration, but precisely because it is more compact and delimited to the cell nucleus. We see CTR2#6 P19 (**a**,**b**), CTR8#14 P12 – RA (**d**,**e**), CTR4#5 P20 (**c**).

**Figure 9:**
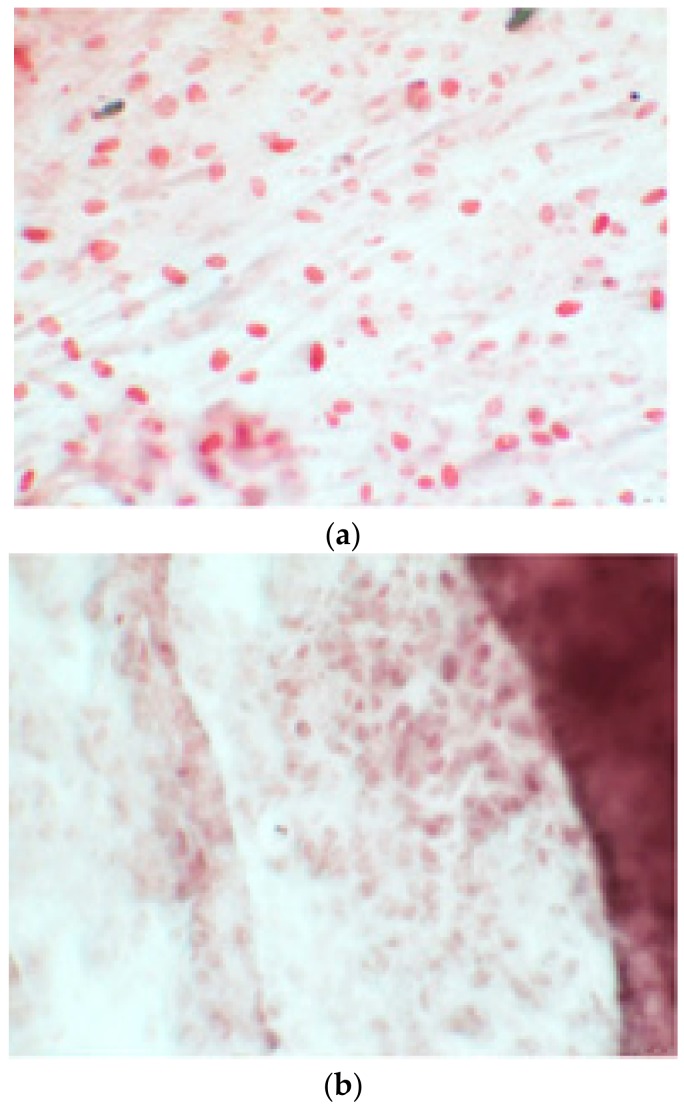

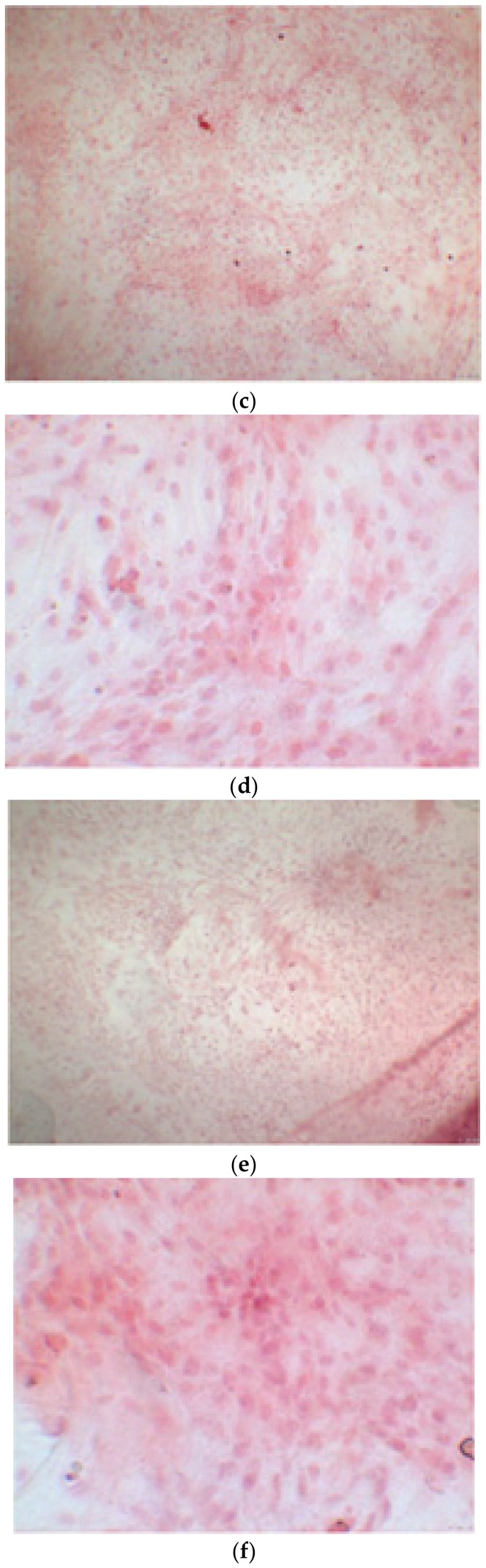
Clone 8 at 28 days showed a much more organized structure, where apparently there seemed to be less staining; this was only due to the fact that the cells, compared to the first test, had a delimited and organized structure (**a–f**).

**Figure 10:**
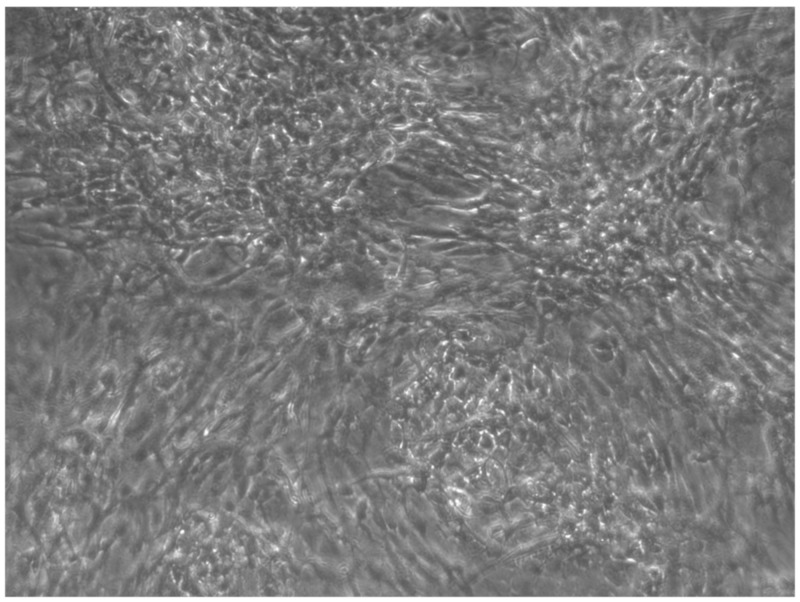
CTR4#5 P19 showed a different structure since the first days of differentiation; this image shows at 28 days how the phase microscope structure was different from the other clones.

The alizarin test was performed at 14, 21, and 28 days depending on the number of cells available for each clone. In the first images processed under the microscope after staining with alizarin, we observed that the clones were positive for the alizarin test after 14 days, so the partially differentiated cells included calcium (Figure 7a–e). Even CTR4#5 P20, which had a different coloring from the others, showed areas of positivity at 14 days (Figure 8). At 21 days, positivity was maintained, as can be seen from Figure 9a–e. In particular, CTR8#14 P12 at 28 and 35 days exhibited a much more organized structure with less staining, but this was only due to the cells, compared with the first test, having a limited and organized structure (Figure 10).

### 3.2. Alkaline Phosphatase

**Figure 11:**
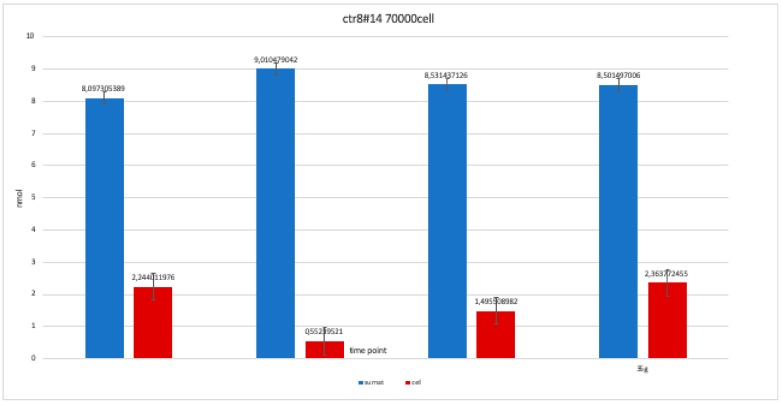
Level of alkaline phosphatase contained in clone CTR8#14 P12 – RA plate on 70,000 cells for the well; the trend is not increasing but rather balanced over time. The clone is the only one to have an alkaline phosphatase of 35 days because it was the clone for which the biological trend developed first, and that produced the largest number of cells, so there wasa greater availability of wells of the plate to carry out tests.

**Figure 12:**
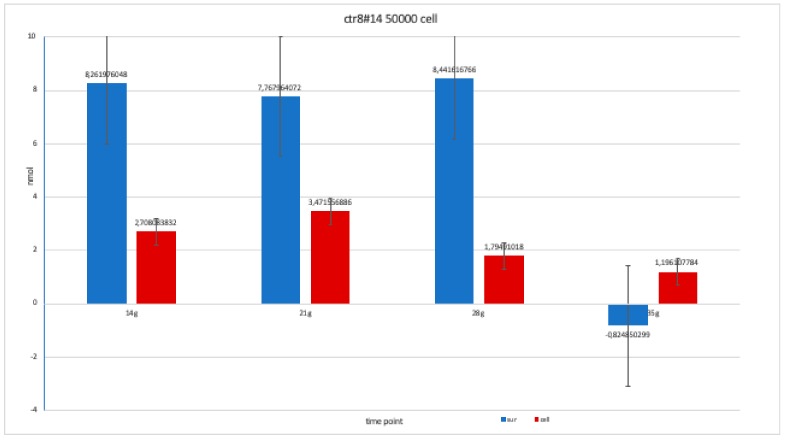
Level of alkaline phosphatase contained in clone in clone CTR8#14 P12 – RA, plated on 50,000 cells for well, showing a fairly balanced trend, except at 35 days when there was an error in the collection of the soil; therefore, this is a bias due to the operation itself.

**Figure 13:**
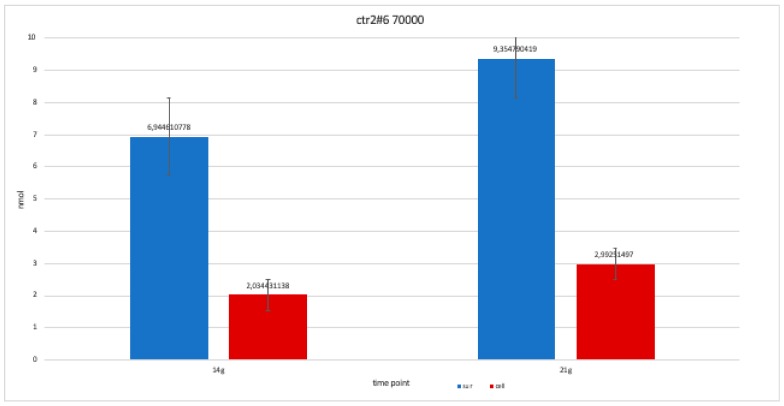
For clone CTR2#6 P19, we only performed two alkaline phosphatases test at 14 and 21 days, since the availability of the number of cells was limited. The trend is increasing but still balanced.

**Figure 14:**
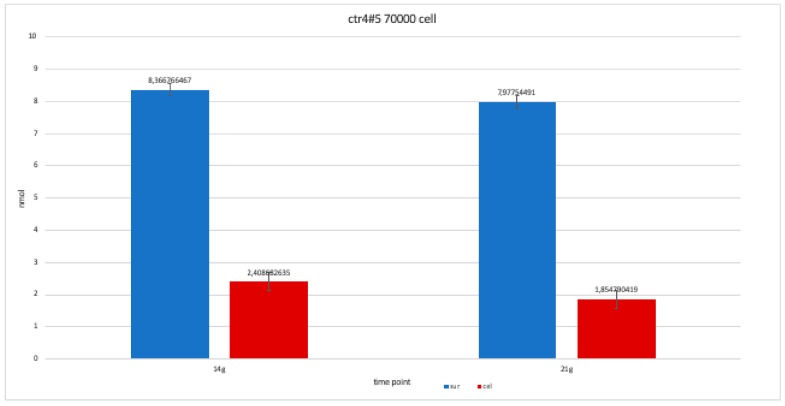
For clone CTR4#5 P20, we only performed two alkaline phosphatases test at 14 and 21 days, since the availability of the number of cells was limited. The trend results are balanced.

**Figure 15:**
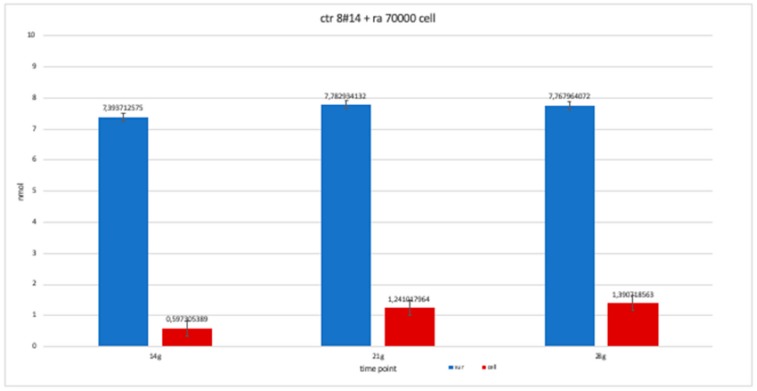
The trend of clone + RA has been studied for the availability of 14, 21 and 28 day cell numbers. The graph is more similar to clone 8-ra, with a trend that is more constant over time than clone 2 and 4.

The nanomolar concentration analyzed in the graphs (Table 2) showed that staining was positive for ALP after 14 days in all clones; this was maintained at 21 and 28 days without changes in trends (Figure 11, Figure 12, Figure 13, Figure 14 and Figure 15).

## 4. Discussion

The ability of induced pluripotent stem cells (HiPS) to be reprogrammed from human tissue and to differentiate into the three embryonic sheets was discovered by Yamanaka in 2006 [17]. Several studies mentioned the reprogramming of cells in the laboratory from cells of oral origin, which have proliferative potential [18,19,20]. Therefore, this type of stem cell has potential for use in personalized medicine in terms of tissue engineering applied to the regeneration of oral tissues; in practical terms, this means stem cells could be combined with scaffold, biomaterials, and/or nanomaterials and growth factors to regenerate alveolar bone [21].

HiPS require further exploration; these types of cells are not known to be similar to embryonic stem cells or cells obtained from amniotic fluid [22]. In addition to the characteristics mentioned above, a trend that emerged in the literature about HiPS is that they are easily accessible, biocompatible, and free from ethical problems, unlike ES cells, and could provide an unlimited source of cells for tissue regeneration [23,24]. However, HiPS can maintain their epigenetic memory, which limits their capacity for proliferation, and they behave unpredictably compared with other clones; they can proliferate uncontrollably to form teratomas, even if this is a process that is tested before conducting experiments. Finally, other concerns reported in the literature are related to HiPS being reprogrammed and then representing an artificial pluripotency, especially when reprogrammed by a virus [25].

One of our findings is that all four clones we tested showed a similar trend: all HiPS were differentiated into osteoblast cells. Clone 4 showed a less regular structure than the others (Figure 10) however, it was positive in the ALP and alizarin red test we conducted. The clones differed in osteoblasts, with a relatively simpler modulated terrain to that described in the literature. Jiang et al. reported the addition of Emdogain® (Botiss biomaterials GmbH, 15806 Zossen, Germany) during osteodifferentiation to accelerate cell growth and proper functioning [14]; however, in our study, the four clones reached full organization in 28 days. Starting from the first days of differentiation, the bone medium without Emdogain® showed the typical lamellar structure of the organization of osteoblasts.

Another variance in our protocol is the use of retinoic acid in control 8 (the clone with the largest cell representation [16]). In our study, clone 8 + RA initially showed a more accelerated trend than clone 8 − RA, but a less orderly organization and a slower trend. Thus, despite its use in standard protocols [14], our protocol produced better results without its use.

Further in vitro testing is needed to apply further changes in terms of the control line and modalities of the experiments conducted, and the second phase of our project will include the use of biomaterials and scaffolds combined with growth factors. A scaffold with an adequate porosity is more biocompatible with bone cells; therefore, it provides a better method of establishing osteoblast cells [26]. The porosity of the biomaterials can compromise the stability of the scaffold [27]. Therefore, once our clones have achieved good results in bone differentiation, we aim to study the biomaterial or nanomaterial that constitutes the ideal scaffold for our cells, perhaps with a three-dimensional design [28].

## 5. Conclusions

Considering the limitations of the present study, osteogenic differentiation from HiPS is a predictable protocol, and in future studies, we will evaluate its potential application in nanomaterials in dentistry. In fact, after in vivo analysis, a biomaterial could be considered as a scaffold to get to the regeneration of the alveolus.

## Figures and Tables

**Table 1:** Number and types of clones used for in vitro tests of the protocol. Type of differentiation (embryoid bodies (EB), or Ooteoblasts (OBs)) were reported, so as alizarin red and alkaline phosphatase (ALP) analysis and timing.

Type of Clone	Differentiation in EB	Differentiation in OBs	Alizarin Red Test/ALP - Timing
CTR2#6 P19	Yes	Yes	Yes - 14, 21, 28 days
CTR4#5 P20	Yes	Yes	Yes - 14, 21, 28 days
CTR8#14 P12 + RA	Yes	Yes	Yes - 14, 21, 28 days
CTR8#14 P12 - RA	Yes	Yes	Yes - 14, 21, 28 days

**Table 2:** This table shows the values obtained from a percentage of alkaline phosphatase (ALP %) obtained with an average of the values at the various observation times (14, 21 and 28 days).

Type of Clone	Number of Cells of Each Clone	Component Where ALP % Was Calculated	14 Days	21 Days	28 Days
ctr8#14	70,000 cells	Supernatant	8,097,305	9,010,479	8,531,437
		Cell	2,244,012	552,395	1,495,509
ctr 8#14	50,000 cells	Supernatant	8,261,976	7,767,964	8,441,617
		Cell	2,708,084	3,471,557	179,491
ctr2#6	70,000 cells	Supernatant	6,944,611	935,479	*-*
		Cell	2,034,431	2,992,515	*-*
ctr4#5	70,000 cells	Supernatant	8,366,766	7,977,545	*-*
		Cell	2,408,683	185,479	*-*
ctr8#14+ ra	70,000 cells	Supernatant	7,393,713	7,782,934	7,767,964
		Cell	0,597,305	1,241,018	1,390,719
